# Obesity-induced extracellular vesicles proteins drive the endometrial cancer pathogenesis: therapeutic potential of HO-3867 and Metformin

**DOI:** 10.1038/s41388-024-03182-2

**Published:** 2024-10-16

**Authors:** Takahiko Sakaue, Kalpana Deepa Priya Dorayappan, Roman Zingarelli, Wafa Khadraoui, Muralidharan Anbalagan, John Wallbillich, Balazs Bognar, Ross Wanner, Casey Cosgrove, Adrian Suarez, Hironori Koga, G. Larry Maxwell, David M. O’Malley, David E. Cohn, Karuppaiyah Selvendiran

**Affiliations:** 1 Division of GYN/ONC, The James Comprehensive Cancer Center, Ohio State University, Columbus, OH USA; 2 Division of Gastroenterology, Department of Medicine, Kurume University School of Medicine, 67 Asahi-machi, Kurume, 830-0011 Japan; 3 Tulane University, New Orleans, LA USA; 4 Institute of Organic and Medicinal Chemistry, Medical School, University of Pécs, Pécs, Hungary; 5 Department of Pathology, The Ohio State University, Columbus, OH USA; 6 Inova Women’s Service Line and the Inova Schar Cancer Institute, Falls Church, VA USA

**Keywords:** Cancer, Ovarian cancer

## Abstract

Endometrial cancer (EC) is the leading gynecologic malignancy in the United States with obesity implicated in 57% of cases. This research investigates the molecular complexities of extracellular vesicles (EV) secretion as carriers of oncogenic protein and their involvement in obesity-mediated EC. An understanding of these mechanisms is pivotal for unraveling pathways relevant to obesity-associated EC, thereby guiding the development of innovative prevention and treatment strategies. Our exploration revealed a significant increase in EV secretion carrying oncogenic proteins (TMEM205, STAT5, and FAS) in adipose and uterine tissues/serum samples from obese EC patients compared to control (without cancer). We identified alterations in EV-regulating proteins (Rab7, Rab11, and Rab27a) in obesity-mediated EC patients, adipose/uterine tissues, and serum samples. Through a 24-week analysis of the effects of a 45% kcal high-fat diet (HFD) on mice, we observed increased body weight, increased adipose tissue, enlarged uterine horns, and increased inflammation in the HFD group. This correlated with elevated levels of EV secretion and increased expression of oncogenic proteins TMEM205, FAS, and STAT5 and downregulation of the tumor suppressor gene PIAS3 in adipose and uterine tissues. Furthermore, our study confirmed that adipocyte derived EV increased EC cell proliferation, migration and xenograft tumor growth. Additionally, we identified that the small molecule inhibitors (HO-3867) or Metformin inhibited EV secretion in vitro and in vivo, demonstrating significant inhibition of high glucose or adipocyte-mediated EC cell proliferation and a reduction in body weight and adipose tissue accumulation when administered to HFD mice. Moreover, HO-3867 or Metformin treatment inhibited HFD induced hyperplasia (precursor of EC) by altering the expression of EV-regulated proteins and decreasing oncogenic protein expression levels. This study provides critical insights into the mechanisms underpinning obesity-mediated EV secretion with oncogenic protein expression, shedding light on their role in EC pathogenesis. Additionally, it offers pre-clinical evidence supporting the initiation of novel studies for EV-targeted therapies aimed at preventing obesity-mediated EC.

## Introduction

Endometrial cancer (EC) is the most common gynecologic malignancy in developed countries and ranks as the sixth most common cancer among women globally [1, 2]. The recent rise in EC cases is linked to the growing obesity epidemic in the developing world. A recent meta-analysis demonstrated a significant increase in EC risk with each 5 kg/m^2^ increase in body mass index (BMI)(risk Ratio (RR) 1.59, 95% confidence interval (CI) 1.59–1.68), particularly for type 1 endometrial cancers. Women with class III obesity with a BMI ≥ 40 kg/m^2^ face an increased lifetime risk of development of developing cancer to 10–15% when compared to 2% in the overall general population [3]. Obesity also influences oncologic outcomes and is association with doubling the risk of cancer-specific mortality [4]. These associated morbidities highlight the importance of understanding the role of obesity-related biomarkers role in cancer development and the potential of possible screening.

Obesity raises the risk of EC in both pre-menopausal and post-menopausal individuals through multiple mechanisms. An increase in the amount of adipose tissue leads to higher circulating estrogen levels by conversion of androgens by aromatase, thereby increasing endometrial cell growth and gene transcription [5]. This effect is more pronounced in the absence of progesterone in an ovulatory state or in a post-menopausal state. Chronic inflammation is another consequence of excess visceral fat and results in various issues like high insulin levels, elevated blood sugar, and reduced anti-inflammatory cytokines. Some proposed mechanisms involve changes in how fat tissue functions, causing alterations in the secretion of adipokines and extracellular vesicles (EV) [6, 7]. Recent studies indicate that obese individuals’ fat cells release EV containing proteins that may raise cancer risk [8, 9].

Extracellular vesicles (EV) are tiny vesicles (30–120 nm) released by various cells and those from adipose tissue play a crucial role in communication between adipocytes and nearby cells [10–12]. However, it has not been clearly elucidated how obesity-related signaling pathways involving EV secretions might affect the development of atypical endometrial hyperplasia and subsequent progression to EC. Our current study has identified the regulation of EV secretion and the expression of oncogenic proteins, specifically TMEM205, STAT5, and FAS, in obesity-associated EC tissues and serum. Furthermore, these proteins (TMEM205, STAT5, and FAS) are overexpressed in EC samples according to the TCGA dataset and published report [13, 14]. Elucidating the molecular mechanisms underlying obesity-mediated EV secretion and oncogenic protein expression in the context of obesity-associated EC is essential for developing alternative strategies for prevention, early biomarkers and therapeutic targeting of EC, including drug-resistant tumors.

We have developed various small molecule inhibitors for targeting ovarian and EC. In this study, we used one such anti-cancer compound, diarylidenyl-piperidone (DAP-HO-3867), to target EV secretion and oncogenic proteins. The HO-3867 compound has a core structure combined with an N-hydroxypyrroline (-NOH) component, which can convert into a nitroxide. HO-3867 compounds exhibit a unique pattern of toxicity: they selectively target cancer cells while sparing healthy cells [15, 16]. This selective toxicity is due to the -NOH group, which protects healthy tissues from damage, while the compound remains toxic to cancer cells, making it a promising therapeutic agent. These compounds are effective at inhibiting STAT3 and FAS, key factors in our research [16, 17]. Additionally, HO-3867 and Metformin have demonstrated significant efficacy in combating EC in a high-fat diet-induced model and in EC cells. These findings suggest that blocking EV secretion pathway proteins and EV proteins using HO-3867 or Metformin could provide a safe and targeted treatment for obesity-related EC.

## Results

### Identified the extracellular vesicle secretion and regulated proteins in obesity associated EC patient samples

**Fig. 1:**
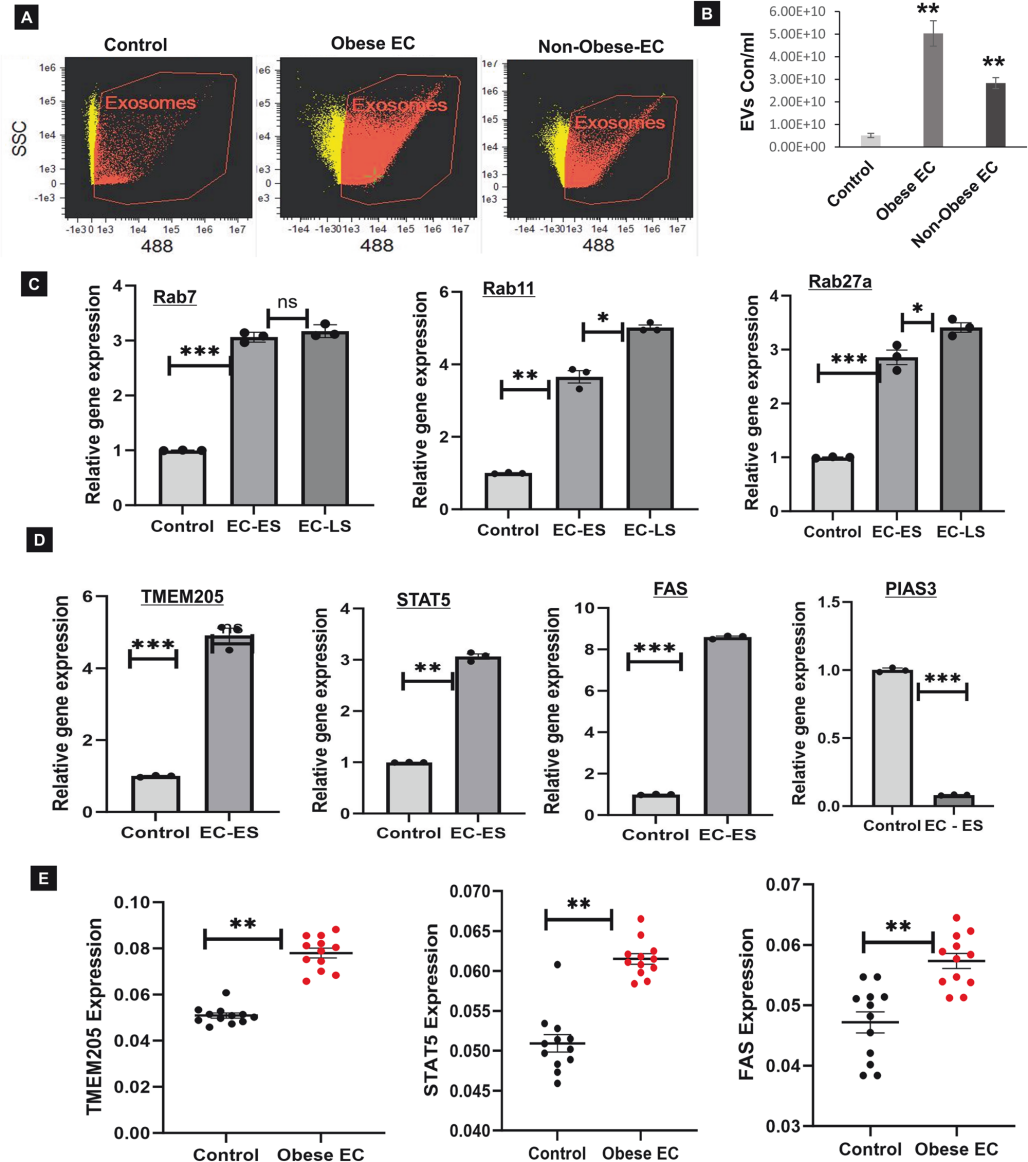
Enhanced extracellular vesicle (EV) secretion associated with oncogenic proteins and EV regulatory proteins in endometrial cancer (EC) patient samples. **A**, **B** Serum samples from control (without cancer), obese EC and non-obese EC patients were thawed on ice and diluted (1:4 to 1:10) in 1xPBS. EV were isolated from 100  µL of patient serum using the q-izon column. EV concentration was assessed by Image Stream Flow Cytometry (ISF), revealing a significant increase in EV concentration and fold change in obese EC patients compared to non-obese EC (*n* = 5, *p* < 0.01). **C** EV **r**egulatory proteins, including Rab7, Rab11, and Rab27a, were analyzed by RT-PCR in control (obese benign) obese EC early-stage (EC-ES) and obese EC late-stage (EC-LS) tissue samples (*n* = 3, *p* < 0.001 or 0.005). **D** Oncogenic proteins (TMEM205, STAT5, FAS, and PIAS3) and their relative gene expression levels were examined in control (obese benign) and EC-ES patient tissues samples by RT-PCR (*n* = 3, p < 0.001 or 0.005). **E** Upregulated TMEM205, STAT5 and FAS protein expression levels in obese EC compare with control (without cancer) patient serum EV samples by ELISA assay (*n* = 12, *p* < 0.005).

In previous studies, EV have been recognized as critical players in cancer progression and metastasis [18–20]. Our research focused on characterizing EV secretion, quantifying their levels, and identifying key proteins involved in their secretion pathways in EC, particularly in patients with obesity-related EC. Using advanced Image Stream Flow Cytometry Analysis (ISA), our findings demonstrated a significant increase in EV release in the serum of obese EC patients compared to non-obese EC patients or control (without cancer) (Fig. 1A, B). Additionally, we examined protein expression profiles related to EV secretion pathways and noted a substantial upregulation of proteins Rab7, Rab11, and Rab27a in obese EC early stage (EC-ES) and EC late-stage (EC-LS) patients’ tissue samples compared to obese benign tissues (Fig. 1C). Furthermore, our investigation uncovered distinct protein expression patterns in obesity-related EC which includes elevated levels of oncogenic proteins such as TMEM205, STAT5, FAS and identified downregulation of tumor suppressor protein PIAS3 (Fig. 1D). In addition, we observed that the oncogenic candidate proteins (TMEM205, STAT5, and FAS) are significantly expressed in the serum EV of obese EC patients compared to control serum EV samples (Fig. 1E). These findings underscore the pivotal role of increased EV levels in obesity-related EC and highlight specific protein expression changes in affected patients. Furthermore, these results suggest that serum EV proteins could serve as early biomarkers and offer therapeutic opportunities for individuals dealing with obesity-related EC.

### Determining the supplementation of high fat diet (HFD) induced endometrial hyperplasia through the EV secretion and alteration of target proteins

**Fig. 2:**
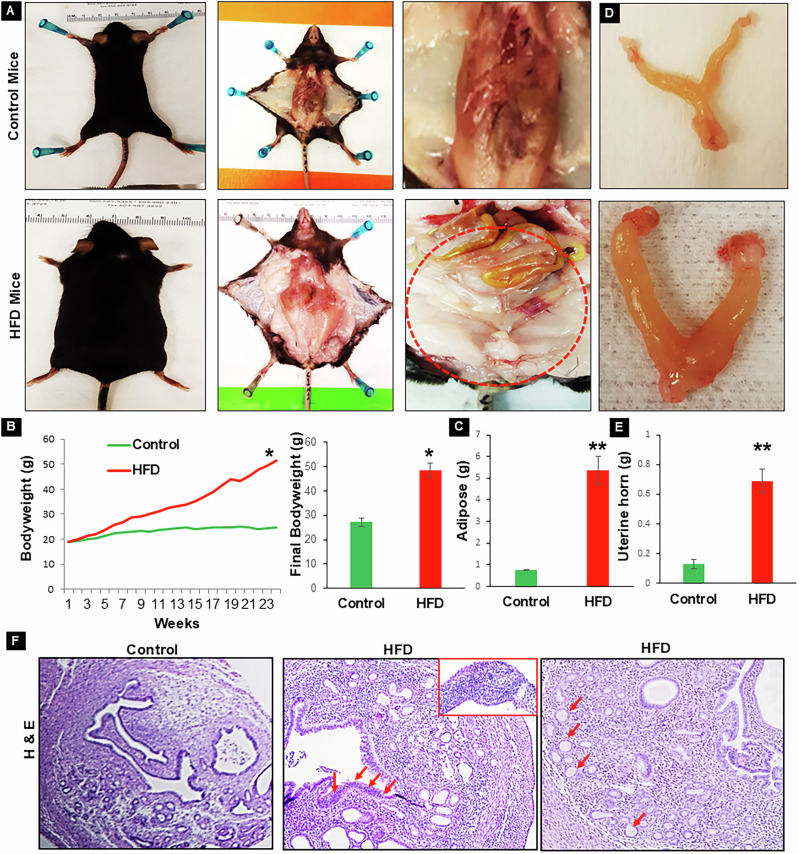
High-Fat Diet (HFD) supplementation promotes adipose tissue accumulation and induces endometrial hyperplasia. **A**, **B** Immunocompetent mice were treated with a high-fat diet (HFD, 45% protein calorie) for 24 weeks. Representative images depict accumulated levels of adipose tissue (circled) in mice fed with a HFD for 24 weeks. Changes in body weight were assessed at the beginning (8 weeks) and end (24 weeks) of the HFD treatment (*n* = 10/group, *p* < 0.01). **C** Increased accumulation of adipose tissue was observed in mice fed with a HFD compared to controls (*n* = 5, *p* < 0.005). **D**, **E** Increased uterine enlargement and weight were observed in mice fed with a HFD compared to controls (*n* = 5, *p* < 0.005). **F** Enhanced cell proliferation in the endometrium of mice fed a HFD compared to controls (normal diet) is illustrated by hematoxylin-Eosin (H&E) staining. The inset shows a higher magnification of the increased cell proliferation.

To investigate the impact of continuous High-Fat Diet (HFD) consumption (45 kcal% fat diet) on the secretion of EV and the regulation of oncogenic proteins in adipose and uterine tissues, we initiated our study by assessing body weight and uterine morphology in mice following 24 weeks of HFD treatment. As expected, mice on the HFD exhibited significantly higher body weight and increased adipose tissue deposition compared to the control group on the regular diet (Fig. 2A–C, Supplementary Fig. 1). Notably, the HFD group displayed pronounced enlargement of uterine horn size (hyperplasia) (Fig. 2D, E), accompanied by observable inflammation and increased cell proliferation in the endometrial layer (Fig. 2F & Supplementary Fig. 2).

**Fig. 3:**
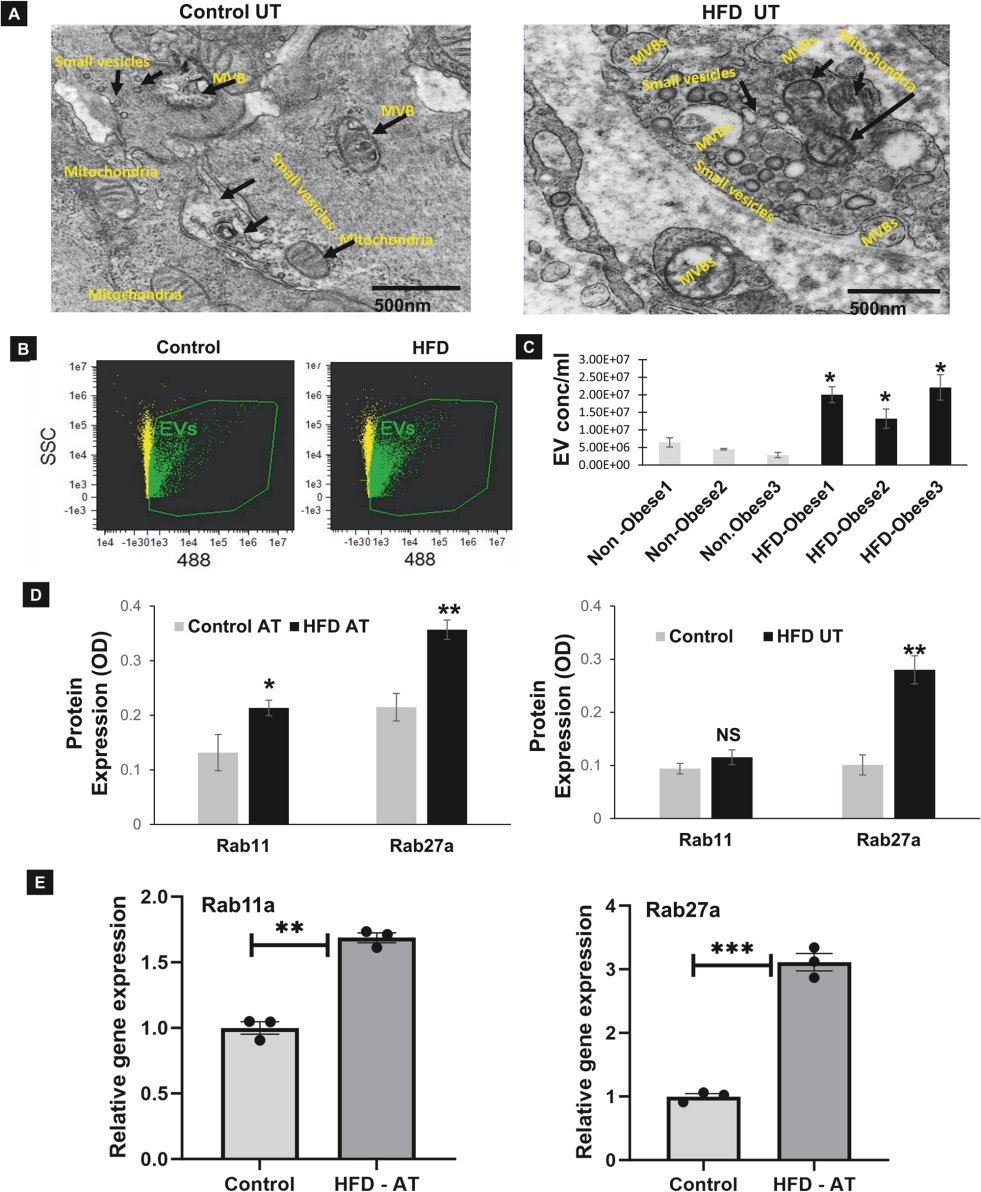
Increased extracellular vesicle (EV) secretion associated with EV regulatory proteins in HFD treated mice samples. **A** Transmission Electron Microscopy (TEM) reveals increased formation of EV in uterine tissues of high-fat diet (HFD) treated as compared to control diet mice. **B**, **C** Quantification of EV in serum samples from control and HFD-treated obese mice using Image Stream analysis (*n* = 3, p*0.01). **D**, **E** Analysis of relative protein and gene expression of EV regulating proteins (Rab11 and Rab27a) levels in adipose and uterine tissues from HFD and control diet-treated mice assessed by ELISA and RT-PCR (*n* = 3, p*0.001 or 0.005).

Subsequently, we explored whether the HFD had an impact on the EV secretion phenotype in uterine tissues. At the 24-week mark, we sacrificed mice from the HFD group and conducted Transmission Electron Microscopy (TEM) analysis. Our observations revealed an increase in EV secretion and the formation of multi-vesicular bodies in uterine tissues from the HFD-treated mice (Fig. 3A, Supplementary Fig. 3), significant elevation of EV secretion was observed in HFD mice serum samples (Fig. 3B, C, Supplementary Fig. 4). Furthermore, there was an upregulation of key EV regulation genes, including Rab11 and Rab27a in HFD treated adipose and uterine tissues (Fig. 3D, E).

**Fig. 4:**
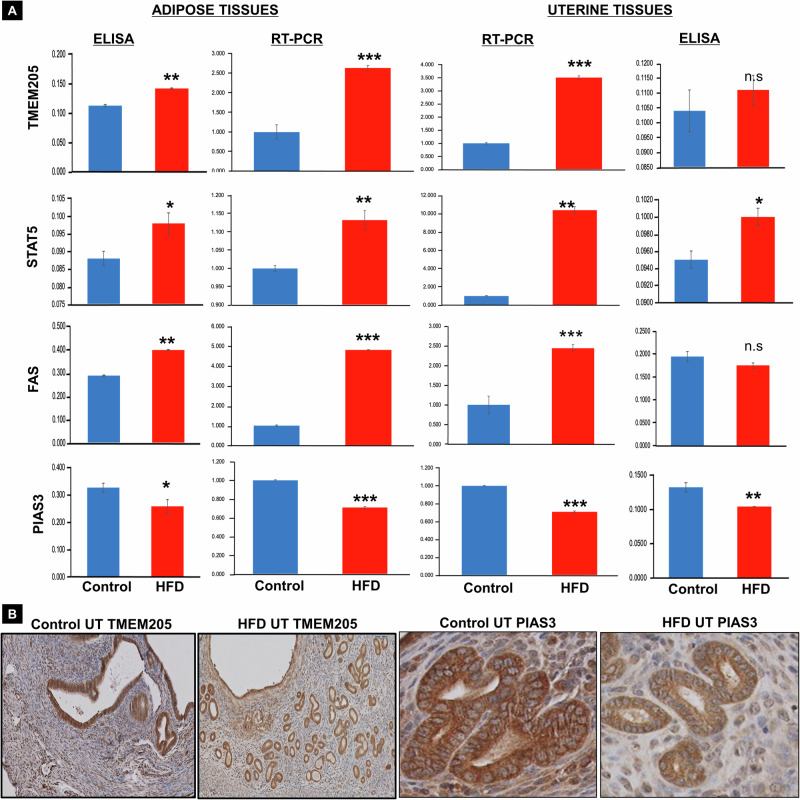
Alterations in oncogenic and tumor suppressor proteins in HFD treated mice tissues. **A**, **B** Analysis of targeted proteins (TMEM205, STAT5, FAS, and PIAS3) and their relative gene and proteins expression levels in adipose and uterine tissues of HFD and control diet-treated mice. The assessment was conducted using a combination of RT-PCR, ELISA, and Immunohistochemistry (IHC) techniques (*n* = 3, *p* < 0.01 or 0.005).

Additionally, our investigation unveiled that HFD supplementation was associated with altered EV secretion patterns and the expression of oncogenic proteins (specifically TMEM205, FAS, and STAT5) and tumor suppressor protein PIAS3. We observed an increased oncogenic proteins and downregulation of the tumor suppressor gene PIAS3 in adipose and uterine tissues (Fig. 4A, B, Supplementary Fig. 5). The cumulative evidence from our study indicates that the changed expression of oncogenic proteins under HFD conditions contributes to cellular proliferation, hyperplasia, and tumor initiation. Consequently, our data strongly suggests that the obese patient tissue microenvironment plays a pivotal role in hyperplasia and tumor initiation of EC through a notable rise in EV release among obese patients with EC. This increase is accompanied by distinction protein expression patterns linked to oncogenic transformation and cancer initiation.

#### Identified the adipocytes derived EV secretion and its role in endometrial cancer

**Fig. 5:**
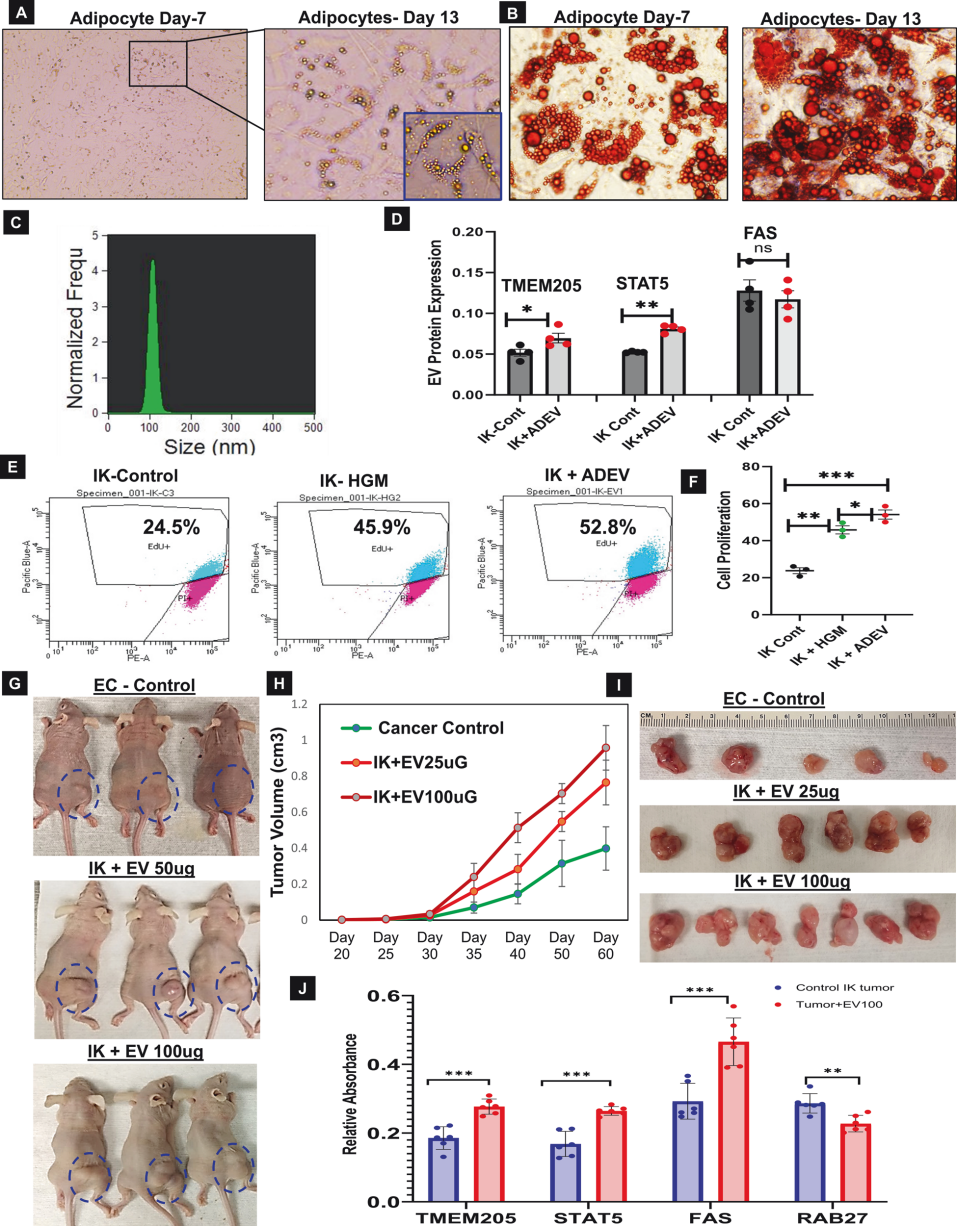
Adipocytes mediated EV secretion increased EC cells proliferation/migration and EC progression. **A** Adipose-derived mesenchymal stem cells underwent adipocyte differentiation initiated 72 h post-confluency on Day 3, 5, and 7, followed by maintenance in adipocyte medium until day 15. Microscopic images (10x and 40x) at different time points show increasing lipid accumulation. **B** To confirm adipocyte formation, cells were cultured in a 12-well plate, fixed, and stained with 0.2% oil red O in 2-propanol for 10 min at room temperature. Adipogenesis was measured at various time points. **C** Adipocyte-mediated extracellular vesicle (EV) secretion and size confirmation by ISF. **D** Adipocyte-derived EV (ADEV) were co-cultured with IK cells for 48 h, and the expression of candidate proteins TMEM205, FAS, and STAT5 was analyzed by ELISA (*n* = 4, *p* < 0.01 or *p* < 0.005). **E**, **F** Cell proliferation assessed by 5-ethynyl 2´-deoxyuridine (EdU) incorporation using flow cytometry in IK cells co-cultured with ADEV, demonstrating an increased percentage of EdU+ve cells compared to IK control cells (untreated ADEV) (*n* = 3, *p* < 0.05 or 0.01). **G** Ishikawa (IK) cells (2 ×10^6 cells) were injected subcutaneously into the right flank of athymic nude mice. One week later, the mice were randomly divided into three groups: Cancer Control (IK cells only, *n* = 6), EV25µg group (IK cells + EV, *n* = 7), and EV100µG group (IK cells + EV, *n* = 7). Adipocyte-derived EV (25 µg and 100 µg per 100 µL) were injected subcutaneously near the tumors in the EV groups, while physiological saline was injected into the Cancer Control group. Injections were administered twice a week for 4 weeks. **H**, **I** Tumor growth was monitored weekly, and tumor size was measured using a linear digital caliper. Tumor volume was calculated and represented in the graph. **J** At the end of the ADEV treatment, the tumor-bearing mice were sacrificed. Tumor tissues were collected and subjected to ELISA to measure the expression of candidate proteins (TMEM205, STAT5, FAS, and Rab27a) in both the control and AEV-treated groups (*n* = 6; *p* < 0.001).

Scientific evidence demonstrates the regulatory role of adipocytes and adipocyte-mediated signaling pathways in cancer initiation and progression, including obesity-associated EC progression [19, 21–24]. Our current study reveals that EV derived from adipocytes and their mediated signaling pathways play a pivotal role in regulating EC cell proliferation, migration, and cancer progression in an in vivo xenograft mouse model. We observed that the differentiated adipocyte derived extracellular vesicles (ADEVs) were enriched with oncogenic proteins-TMEM205, STAT5 and FAS. (Fig. 5A-D, Supplementary Fig. 6). Co-culturing the ADEVs with EC Ishikawa (IK) cells resulted in a significant increase in EC cell proliferation (Fig. 5E, F and Supplementary Fig. 7A). To further investigate the role of ADEVs in endometrial cancer (EC) xenograft tumor growth, we conducted an experiment using nude mice. IK cells were injected into the mice, and after two weeks, AEV was administered weekly near the xenograft tumors. The results showed a significant increase in EC tumor growth in the ADEV-treated mice compared to the untreated group (Fig. 5G–I). Additionally, oncogenic protein expression analysis revealed that TMEM205, FAS, and STAT5 levels were significantly elevated and Rab27 decreased in the ADEV-treated xenograft tumors (Fig. 5J). These findings suggest that ADEV play a crucial role in EC progression within an obesity context.

### Impact of EV secretion inhibitor compound HO-3867 and Metformin on in vitro and in vivo HFD induced endometrial hyperplasia

**Fig. 6:**
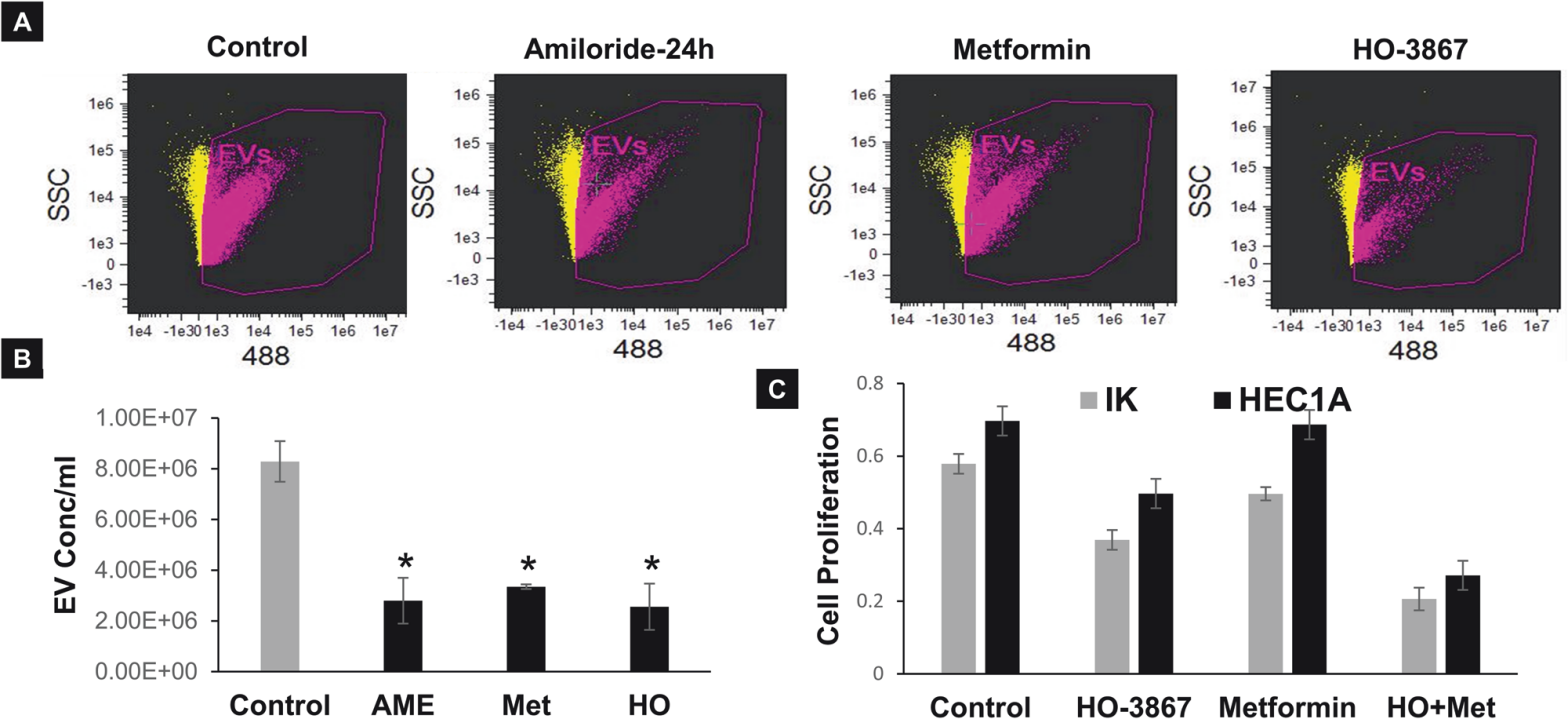
Effects of EV inhibitors on EV secretion in EC cells. **A**, **B** EV secretion levels analyzed and quantified by ISF in EV inhibitors Amiloride (10uM), GW4869 (5uM), Metformin (10uM), or HO-3867 (5uM) for 24 h in EC cells (*n* = 3, *p* < 0.001). **C** Cell proliferation assay measured in IK cells treated with small molecule inhibitors HO-3867 or Metformin for 24 h (*n* = 5, *p* < 0.01).

Our recent investigations have highlighted the impact of high glucose levels on EC cell proliferation, causing alterations in oncogenic proteins and microRNAs [9]. In this study, we aimed to elucidate the effects of high glucose-mediated EV secretion on EC cell proliferation and migration, and to evaluate the inhibition of EV secretion in EC cells. Ishikawa EC cells were cultured in low, normal, or high glucose media for 24–72 h. Observations indicated that high glucose-treated EC cells exhibited enhanced proliferation and migration compared to cells treated with regular glucose (Supplementary Fig. 7B, C). Notably, blocking EV secretion using an established EV inhibitor Amiloride (AME), a small molecule inhibitor (HO-3867), or Metformin resulted in a significant reduction of EV secretion and EC cell proliferation (Fig. 6A–C).

**Fig. 7:**
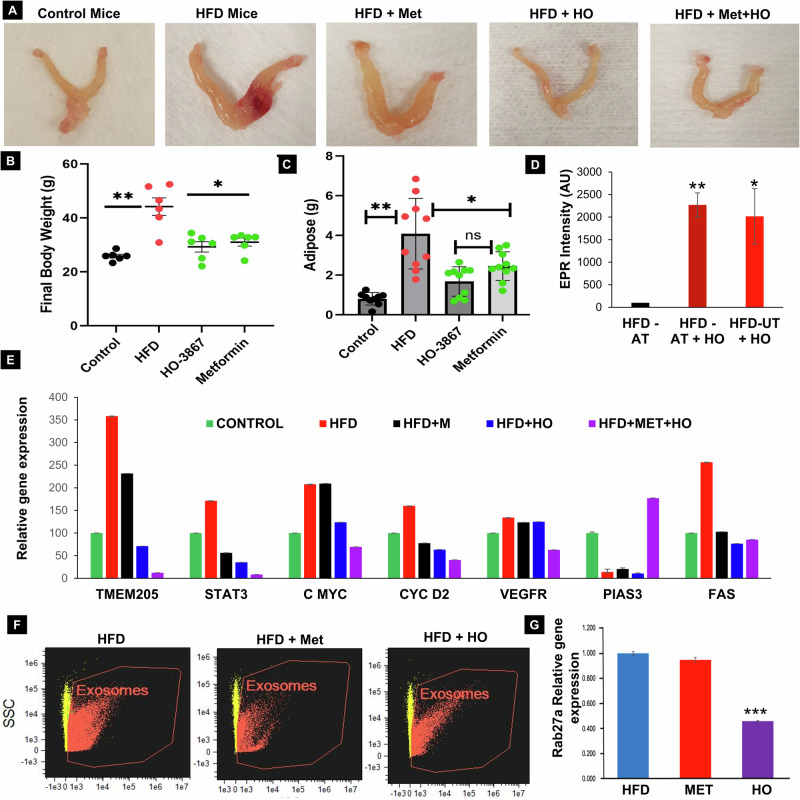
Effects of Metformin and HO-3867 on EV secretion in HFD-Fed Mice: prevention of endometrial cancer. **A** Representative images of uterine tissues in mice fed a high-fat diet (HFD) for 24 weeks, relative to those fed a control diet or treated with HO-3867 (2 mg/kg) or Metformin (5 mg/kg in drinking water or food). **B**, **C** Reduction in body weight and adipose tissue’s accumulation were observed in HFD-fed mice following treatment with HO-3867 or Metformin (*n* = 7 or 10, p*0.01 or 0.001). **D** Electron paramagnetic resonance (EPR) spectra obtained from adipose and uterine tissue biopsies of HFD-treated mice, illustrating the presence of HO-3867 in its oxidized (nitroxide) form. The levels of HO-3867 in the tissue samples were quantified using EPR (*n* = 4; *p* < 0.005). **E** Relative expression levels of target genes determined by RT-PCR in Metformin- or small molecule inhibitor (HO-3867)-treated uterine tissues of HFD-fed mice (*n* = 5, *p* < 0.05). **F**, **G** Isolation of EV from HFD and DAP-HO treated mice serum samples, with quantification of EV secretion levels by Image stream flow cytometry (ISF). EV regulating protein Rab27a was analyzed in Metformin or HO treated HFD mice uterine tissues by RT-PCR (*n* = 3; *p* < 0.001).

Further, we explore the impact of inhibiting HFD-mediated EV secretion and the modulation of oncogenic proteins in adipose and uterine tissues, our study employed a small molecule inhibitor targeting EV regulatory pathway proteins (STAT3 and TMEM205) [18, 25, 26], along with Metformin to inhibit EV secretion, the administration of HO-3867 and Metformin showed a notable trend towards reduced body weights (Supplementary Fig. 9). Importantly, the morphology of uterine, total body weight and adipose tissue accumulations of the mice at the conclusion of the experiment was similar between the treatment and control (untreated) groups (Fig. 7A–C). Furthermore, electron paramagnetic resonance (EPR) spectra intensity measured from adipose and uterine tissue biopsies of HFD-treated mice revealed the presence of HO-3867 in its oxidized (nitroxide) form (Fig. 7D). Endometrial hyperplasia tissues collected were subjected to reverse transcription-polymerase chain reaction (RT-PCR), demonstrating a decrease in the candidate oncogenic gene expression of TMEM205, FAS, STAT5, c-MYC, Cyclin D2, VEGFR, and an increase in tumor suppressor gene PIAS3 in Metformin and HO-3867 combination group versus control mice (Fig. 7E). Additionally, we observed a decreased EV secretion that correlated with reduced Rab27a gene expression upon treatment with HO3867 (Check Fig. 7G) in mice endometrial hyperplasia serum/tissue samples (Fig. 7F, G). These results indicate that both HO-3867 and Metformin effectively decrease the expression of oncogenic proteins and EV regulating proteins to suppress the secretion of EV within the context of HFD-induced endometrial hyperplasia and consequently leading to EC.

## Discussion

In our current research, we have achieved several significant new findings: (i) Obesity-associated endometrial cancer (EC) patients and mice exhibited markedly elevated levels of extracellular vesicle (EV) secretion containing oncogenic proteins in their serum and tissue samples; (ii) We observed upregulation of oncogenic proteins (TMEM205, STAT5, and FAS), as well as the downregulating tumor suppressor protein PIAS3 in samples of adipose and uterine tissues from EC patients and high-fat diet (HFD) mediated obesity mice; (iii) Adipocytes mediated EV secretion play a key role in EC cells proliferation/migration and EC progression; (iv) Our DAP derivative compound HO-3867 and Metformin effectively inhibits blocks EV secretion in both in vitro and in vivo mouse models, which resulted in the inhibition of the growth of obesity-associated EC, both in vitro and in vivo.

Although the link between obesity and the onset of endometrial hyperplasia, progressing to EC [27–29], is widely acknowledged, the specific cellular mechanisms by which obesity influences this progression are not fully elucidated. Various potential pathophysiological mechanisms have been suggested, encompassing alterations in the physiological function of adipose tissue, resulting in chronic inflammation, and changes in the secretion patterns of adipokines and extracellular vesicles [30–32]. Recent investigations have unveiled the role of adipocytes from obese patients in secreting EV carrying oncogenic proteins, thereby increasing the incidence of cancer [8, 9, 19]. EV, originating from adipose tissue, play a pivotal role in mediating communication between adipocytes and neighboring cells [33–35]. Nevertheless, the impact of obesity-mediated signaling pathways involving EV secretion on the development of atypical endometrial hyperplasia progression to carcinoma remains a subject of uncertainty. Our study presents compelling evidence that highlights the critical role of obesity-associated EV carrying oncogenic proteins, including TMEM205, STAT5, and FAS in the pathogenesis of EC. These EV-borne oncogenic proteins have the potential to fuse with adjacent cells, instigating a cascade of events that ultimately result in the downregulation of tumor suppressor protein PIAS3 within both adipose and uterine tissues. This downregulation sets the stage for increased cellular proliferation and endometrial hyperplasia, thus initiating the growth of EC. Previous reports show that in various cancers, including obese cancer patients, increased EV secretion directly corresponds to elevated expression of oncogenic proteins [21, 36, 37]. Our current findings provide valuable insights into how EV-mediated signaling plays a pivotal role in the complex interplay between obesity and the development and progression of EC.

While the oncogenic roles of STAT5, FAS, and TMEM205 have been investigated in various cancers [38–40], their involvement in the regulation of EV secretion and obesity-associated EC remains incompletely understood. Recent study has revealed that STAT3 or TMEM205 co-localizes with EV secretion proteins, specifically Rab8 or Rab11 [18, 41–43], and its expression is upregulated in ovarian cancer cells, where it plays a crucial role in regulating EV secretion. Based on our understanding of the roles of GTPases such as Rab7, Rab11, and Rab27 in vesicle trafficking [44–46], we investigated whether the upregulation of oncogenic proteins (STAT5, TMEM205, and FAS) could lead to their co-localization with Rab7, Rab11, or Rab27. This co-localization may enhance the rate of vesicle recycling and subsequently increase EV secretion in obesity-associated adipose and endometrial tissues. As a result, elevated EV secretion containing oncogenic proteins may contribute to the progression of EC in obese patients. Nevertheless, further studies are warranted to investigate the mechanisms behind the upregulation of TMEM205, STAT5, and FAS and their interactions with Rab family proteins. Specifically, research should focus on how these interactions regulate Rab11 or Rab27a and contribute to increased EV secretion in obesity-associated endometrial cancer.

Previous studies have established that the inhibition of EV secretion using diuretic agents like amiloride (AME) or small molecule inhibitors such as GW4869 can effectively reduce the growth of pancreatic, lung, and colon cancers [47–49]. However, the potential impact of inhibiting proteins involved in EV secretion pathways or blocking EV secretion as a means of prevention or oncotherapy in the context of obesity-mediated EC has not been previously explored. We have developed a small molecule inhibitor, diarylidenylpiperidones-NOH (HO-3867). HO-3867 demonstrates selective inhibition of EV secretion in cancer cells while sparing normal cells. The HO-3867 molecule incorporates an N-hydroxypyrroline (NOH), which serves as a nitroxide precursor, functioning as a cytotoxicity modulator [16]. This unique feature imparts antioxidant protection to noncancerous tissues, making DAP compounds a potentially safe inhibitor for EV regulating oncogenic proteins (STAT3, FAS, and TMEM205), with applications in preventing obesity-mediated EC and other cancers within an obese microenvironment.

Although a consensus on the definitive cancer prevention or anticancer effects of the DAP compound HO-3867 or Metformin in endometrial cancer, both in vitro and in vivo, remains elusive, our current study highlights the importance of targeting EV secretion pathway proteins in obesity-associated EC. Our in vivo investigations using diverse animal models hold promise for substantiating whether the inhibitory effects of HO-3867 or Metformin on the EV secretion pathway significantly contribute to their overall cancer prevention. These upcoming studies may also reveal novel therapeutic applications for inhibiting EV secretion in the ongoing fight against cancer.

## Materials & methods

### Procurement, processing, and handling of human samples

Retrospectively collected EC patient samples were obtained from The Ohio State University James Cancer Hospital and Solove Research Institute (patient samples *N* = 20), (IRB study number – 2022C0070) and Inova Schar Cancer Institute. Patients were assessed by physician review of clinical records at the time of serum collection and were considered to be obese early and late-stage EC before a chemotherapy regimen. De-identified patient information including age (>30–70), BMI (Normal (19–24.9); Overweight (25–29.9); Obese (>30), Waist circumference, EC diagnosis (Stage I/II, III and IV), histology, germline genetics, and disease distribution was obtained by retrospective review of clinical records. For the controls, samples were included if the patient had no documented adnexal pathology, cancer, or comorbidities. The patient serum samples were stored at −80 °C and were subjected to thawing at 30–50 min prior to experiments.

#### Cell lines and cultures

The Ishikawa (grade 1) and HEC-1 endometrial cancer cell lines were used in this study (obtained from ATCC). The cells were grown in DMEM with either low (1 mM), normal (5 mM), or high (25 mM) concentrations of glucose. The media was supplemented with 10% heat-inactivated FBS, 2% sodium pyruvate, 1% penicillin, and 1% streptomycin. Cells were grown in a 75 mm flask to 70% confluence at 37 °C in an environment of 5% CO_2_ and 95% air. Cells were trypsinized (0.05% trypsin/EDTA) routinely. We confirmed mycoplasma activity using ATCC® Universal Mycoplasma Detection Kit in all cells line every 2 months. Once the frozen cells were thawed, they were passaged a maximum of 5 times before being discarded, after which a fresh vial was thawed.

### High fat Diet (HFD) study in immunocompetent mice

C57BL/6 Female immunocompetent mice were procured from the Charles River, MA, USA. The mice were categorized into two groups: the Normal Chow Diet group (NCD group, *n* = 10) and the High Fat Diet group (HFD group, *n* = 12). These mice were age-matched and co-habitated in well-ventilated cages with solid flooring, grouped in 2–3 individuals. They were provided unrestricted access to food and water on a 24-h basis. The NCD group adhered to a normal chow diet throughout the study until reaching 24 weeks of age. The selected diet for the NCD group contained 3% kcal fat content, recognized as the standard diet for research mice. The HFD group, on the other hand, was immediately transitioned to a high fat diet upon arrival at three months of age, maintaining this diet until the conclusion of the study at 24 weeks of age. The high fat diet comprised 45% kcal fat (Research Diets, NJ, USA). At 25 weeks of age, followed by euthanasia and systematic harvesting of animal organs. In the therapeutic study, female mice were stratified into five groups (NCD, HFD, HFD + HO-3867, HFD + Metformin, and HFD + HO + Metformin) for distinct treatments. The treatments commenced after 16 weeks of high fat diet exposure, spanning 8 weeks, after which the mice were sacrificed. Uterine, ovarian, and adipose tissues were collected for subsequent molecular analysis.

### Transmission electron microscopy (TEM)

The uterine and adipose tissues were processed for TEM imaging as follows: The tissues were dissected and fixed in 2.5% glutaraldehyde in 0.1 M phosphate buffer for at least 24 h at 4 °C. Likewise. The adipose and uterine tissues were washed and then fixed with 2.5% glutaraldehyde in 0.1 M phosphate buffer for 30 min at room temperature. Both tissue samples were postfixed with 1% osmium tetroxide and then enbloc stained with 1% aqueous uranyl acetate, dehydrated in a graded series of ethanol, and embedded in Eponate 12 epoxy resin (Ted Pella Inc., Redding, CA). Ultrathin sections were cut with a Leica EM UC6 ultramicrotome (Leica microsystems Inc., Deerfield, IL), collected on copper grids. Images were acquired with an FEI Technai G2 Spirit transmission electron microscope (Thermo Fisher Scientific, Waltham, MA), and a Macrofire (Optronics, Inc., Chelmsford, MA) digital camera and AMT image capture software.

### RNA isolation and reverse transcription PCR (RT-PCR)

Total RNA was isolated from tissues or EC cell samples using the RNeasy Mini Kit (Qiagen, Valencia, CA, USA). RNA samples with an optical density A260/A280 ratio between 1.8 and 2.1 were used. RT‐PCR was then performed using the Transcript First Strand Complementary DNA (cDNA) Synthesis Kit (Roche Diagnostics, Indianapolis, IN, USA) to synthesize cDNA. RT‐PCR was performed with 1 mg of RNA template. The reaction was carried out using the Veriti Thermal Cycler (Applied Biosystems, Thermo Fisher Scientific) and random hexamer primers.

### Isolation of EV using SEC columns

IZON qEV original size exclusion columns (Izon Science) were used in the isolation of EV. The columns were first removed from 4 °C and the 20% ethanol storage solution was allowed to run through the column followed by 20 ml particle-free PBS. Serum samples were diluted to 500 µl with sterile filtered particle-free PBS and the sample was overlaid on the qEV size exclusion column followed by elution with particle-free PBS. The flowthrough was collected in 500 µl fractions, and fractions 1–4 were pooled for further downstream analysis such as Image stream flowcytometry. For ELISA plating the pooled EV samples were further concentrated using an Amicon Ultra-0.5 ml (10 kDa) centrifugal filter device (Merck Millipore) for protein estimation and relative quantification of potential EV biomarker proteins across different patient samples.

### Statistical analysis

Results were expressed as mean ± S.D. Comparisons between groups were made by a Student’s *t* test. The significance level was set at *p* ≤ 0.05.

## Supplementary information

Supplemental Figure 1 to Figure 9

## Data Availability

The datasets utilized and/or analyzed during this study are available from the corresponding author upon reasonable request.
