# Correction: Obesity-induced extracellular vesicles proteins drive the endometrial cancer pathogenesis: therapeutic potential of HO-3867 and Metformin

**DOI:** 10.1038/s41388-026-03949-9

**Published:** 2026-08-03

**Authors:** Takahiko Sakaue, Kalpana Deepa Priya Dorayappan, Roman Zingarelli, Wafa Khadraoui, Muralidharan Anbalagan, John Wallbillich, Balazs Bognar, Ross Wanner, Casey Cosgrove, Adrian Suarez, Hironori Koga, G. Larry Maxwell, David M. O’Malley, David E. Cohn, Karuppaiyah Selvendiran

**Affiliations:** 1 Division of GYN/ONC, The James Comprehensive Cancer Center, Ohio State University, Columbus, OH USA; 2 Division of Gastroenterology, Department of Medicine, Kurume University School of Medicine, 67 Asahi-machi, Kurume, 830-0011 Japan; 3 Tulane University, New Orleans, LA USA; 4 Institute of Organic and Medicinal Chemistry, Medical School, University of Pécs, Pécs, Hungary; 5 Department of Pathology, The Ohio State University, Columbus, OH USA; 6 Inova Women’s Service Line and the Inova Schar Cancer Institute, Falls Church, VA USA

**Keywords:** Cancer, Ovarian cancer

Correction to: *Oncogene* 10.1038/s41388-024-03182-2, published online 16 October 2024

While reviewing data from our previous obesity study for a new grant application, I identified an error in the selection of one representative H&E image used in Figure 2F for control group. I mistakenly interpreted the label “C1” as representing the control group, when in fact it corresponded to a high-fat diet (HFD) study mouse.

Former Figure 2:

**Figure:**
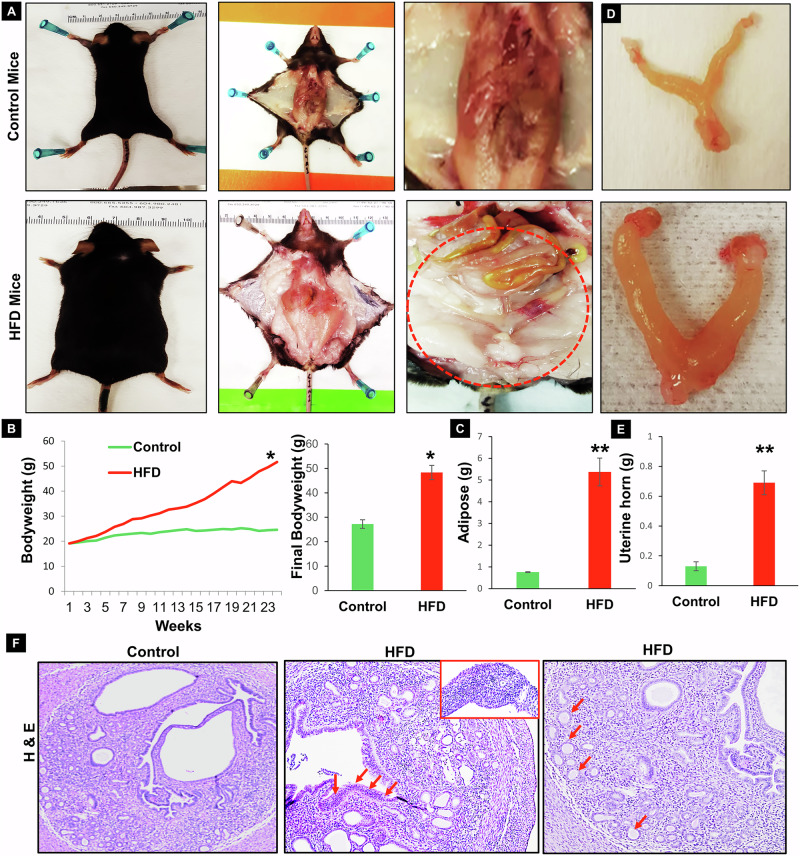

Corrected Figure 2:

**Figure:**
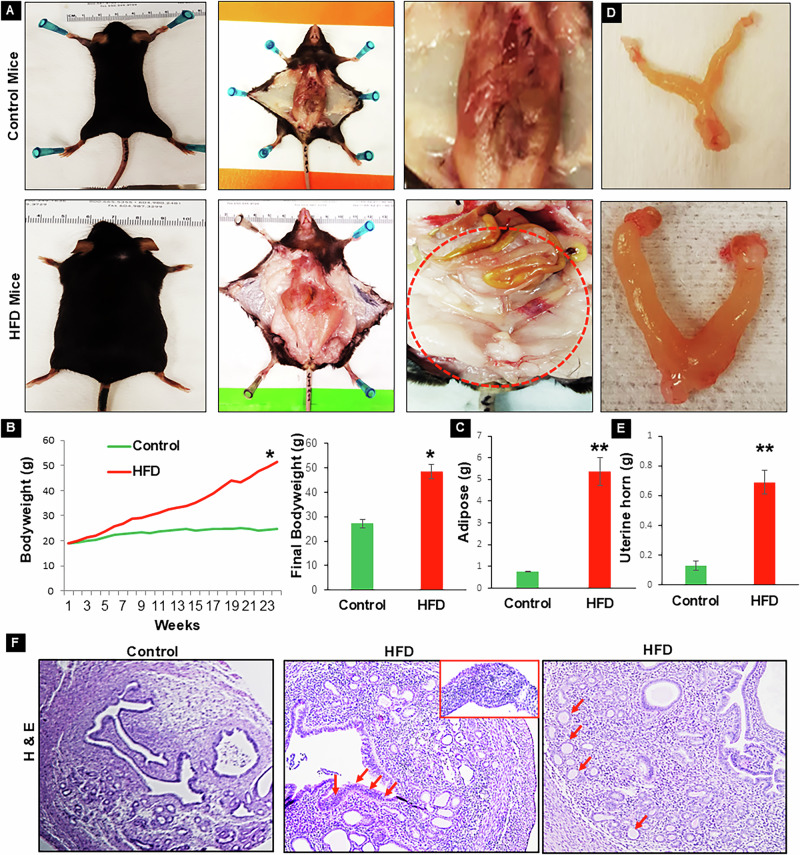

The original article has been corrected.

