# Supplementary material for: Obesity-induced extracellular vesicles proteins drive the endometrial cancer pathogenesis: therapeutic potential of HO-3867 and Metformin
Source: Oncogene. 2024 Oct 16;43(49):3586–97. doi: 10.1038/s41388-024-03182-2 (PMC11602708; doi:10.1038/s41388-024-03182-2)
Supplement: Supplementary file 1 — Supplemental Figure 1 to Figure 9 [file 41388_2024_3182_MOESM1_ESM.pdf]

Sup Figure 1

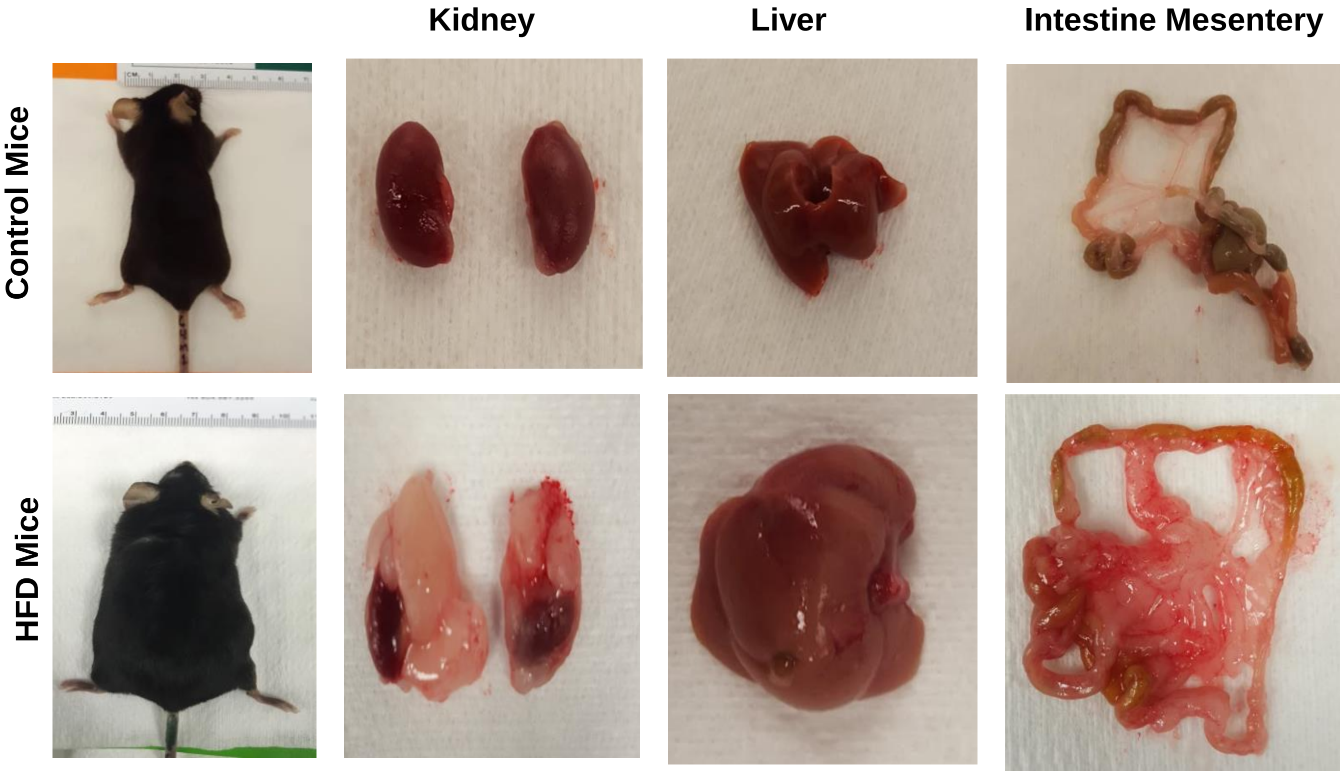

Sup. Fig.1. Immunocompetent mice were treated with a high-fat diet (HFD, 45% protein calorie) for 24 weeks. Changes in mice body and organ weight were assessed at the end (24 weeks) of the HFD treatment (n = 10/group, $p<0.05$).

Sup Figure 2

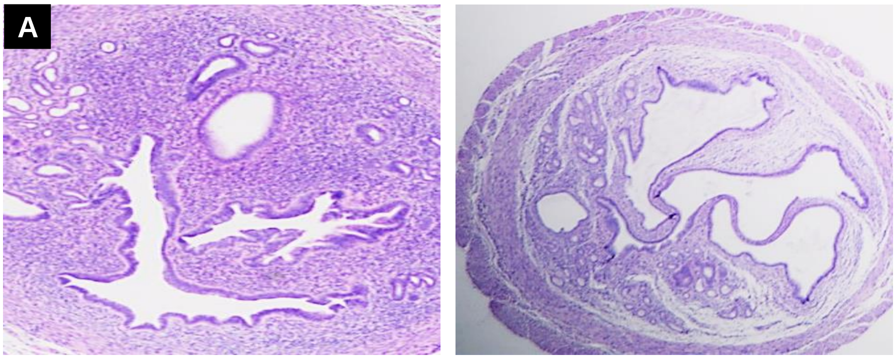

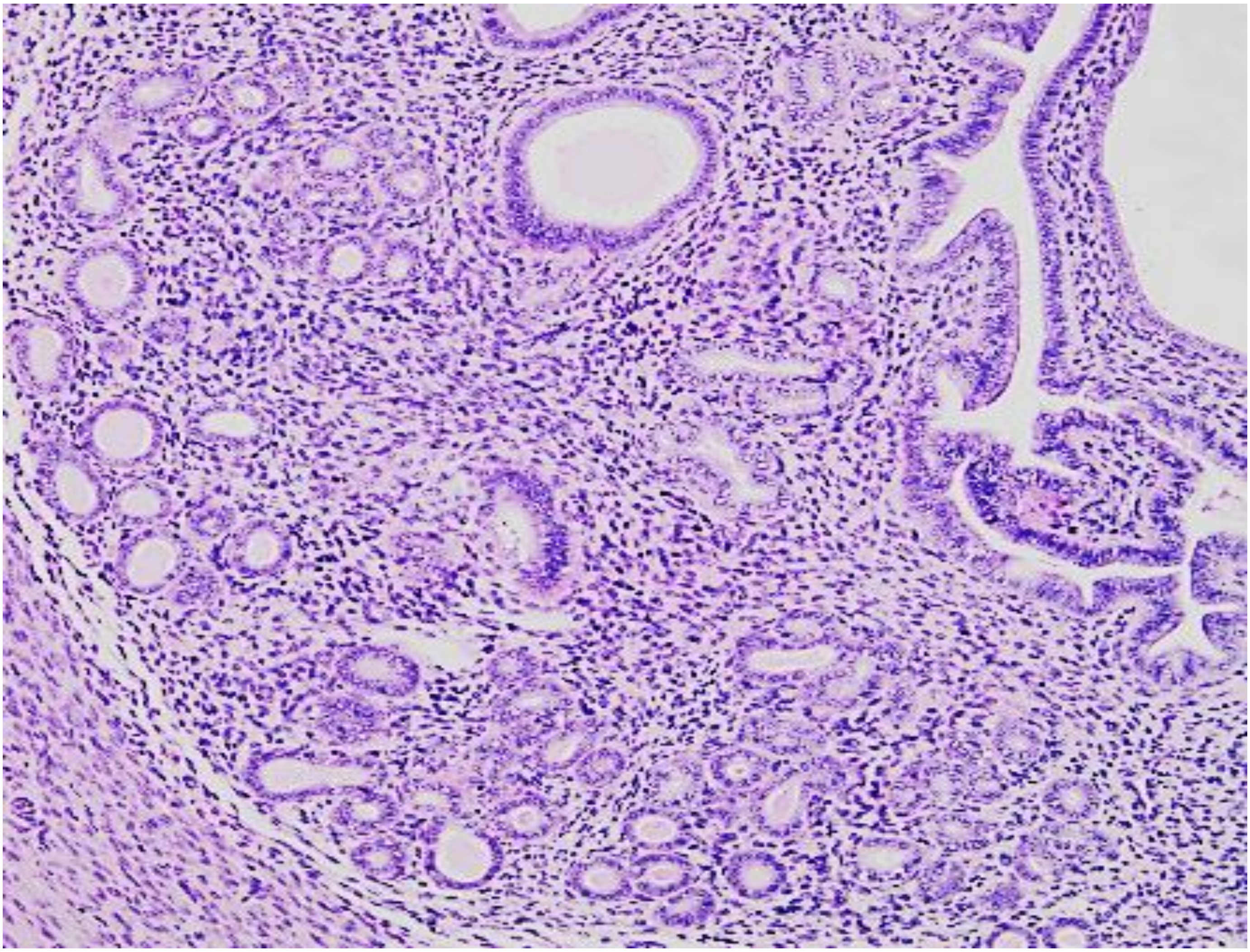

Sup. Fig. 2. A & B. Morphological changes in the uterus, which is enlarged in mice fed a HFD for 24 weeks.

Sup Figure 3

TEM Analysis of vesicles in Obese Mice Control and HFD Uterine tissues

Control Uterine

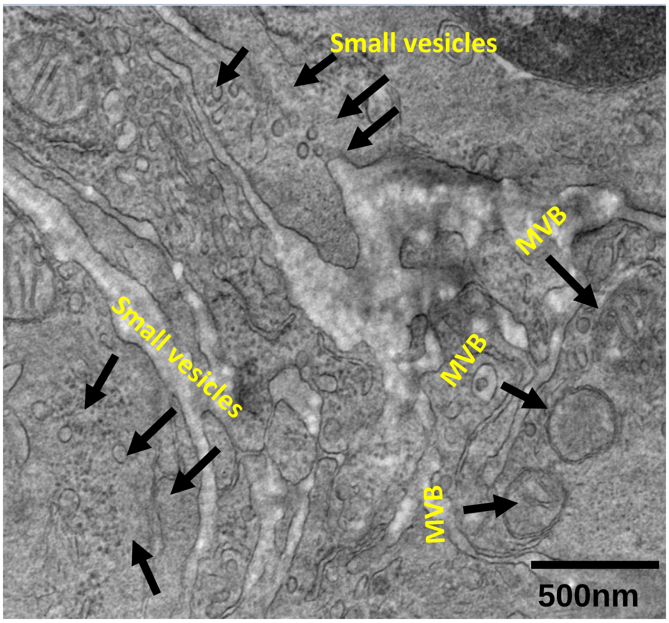

HFD Uterine

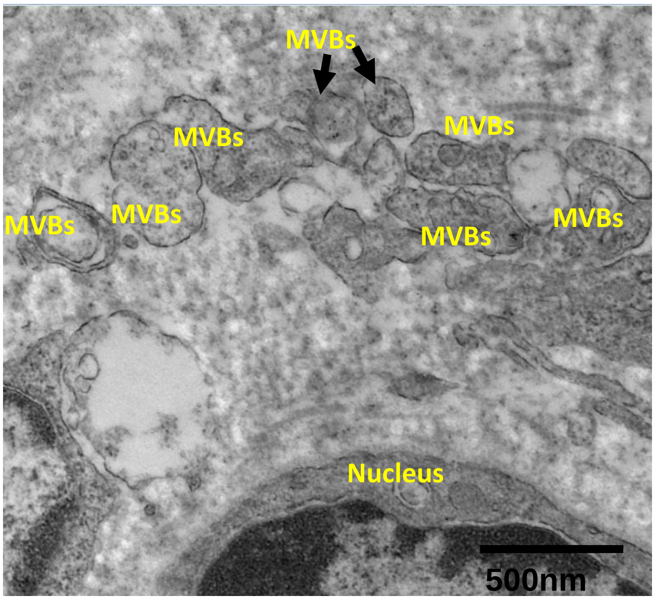

Sup. Fig. 3. Transmission Electron Microscopy (TEM) reveals increased formation of EVs in uterine tissues of high-fat diet (HFD) treated and control diet mice.

Sup Figure 4

<u>**Image Stream analysis of Vesicles from mice serum (Control and Obese)**</u>

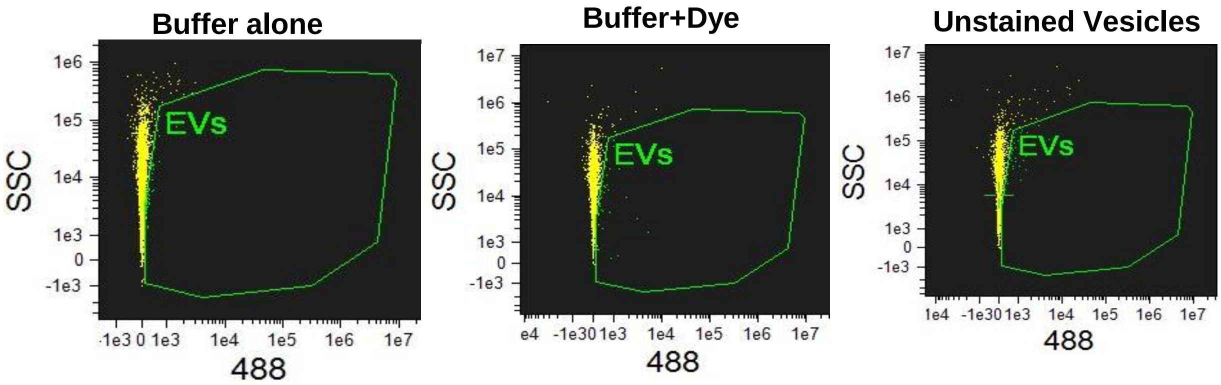

Sup. Fig. 4. Buffer and unstained controls of EV serum samples analyzed by Image stream flowcytometry analysis

Sup Figure 5

Control AT PIAS3

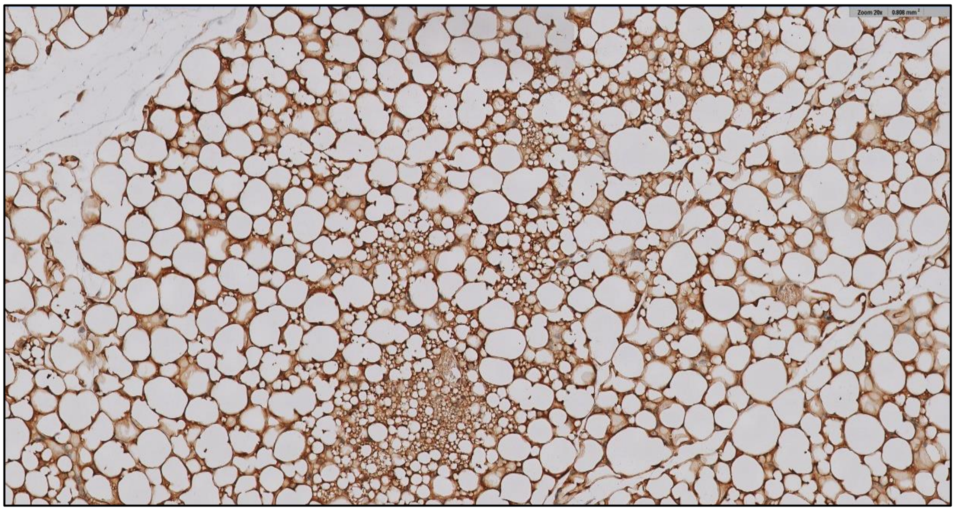

HFD AT PIAS3

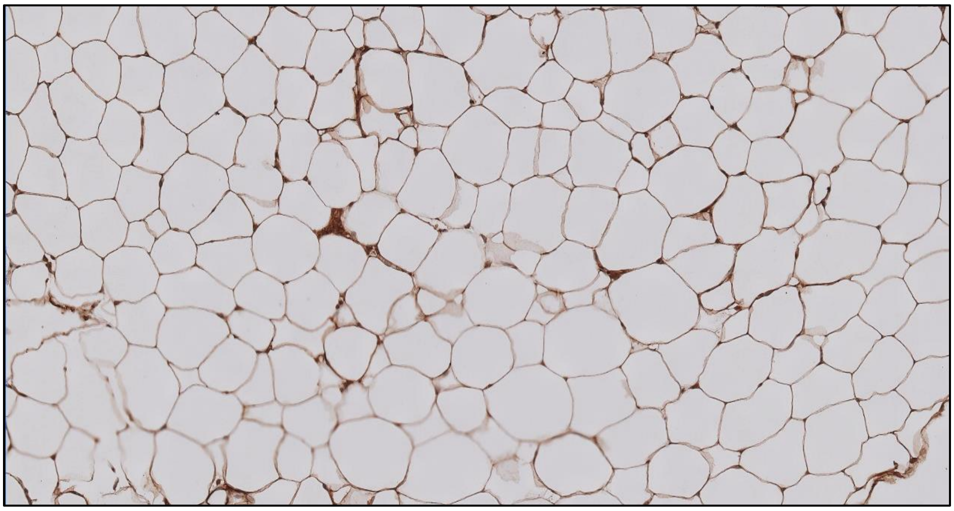

Sup. Fig. 5. PIAS3 expression alteration in HFD treated adipose tissues compared with control diet-treated mice.

Sup Figure 6

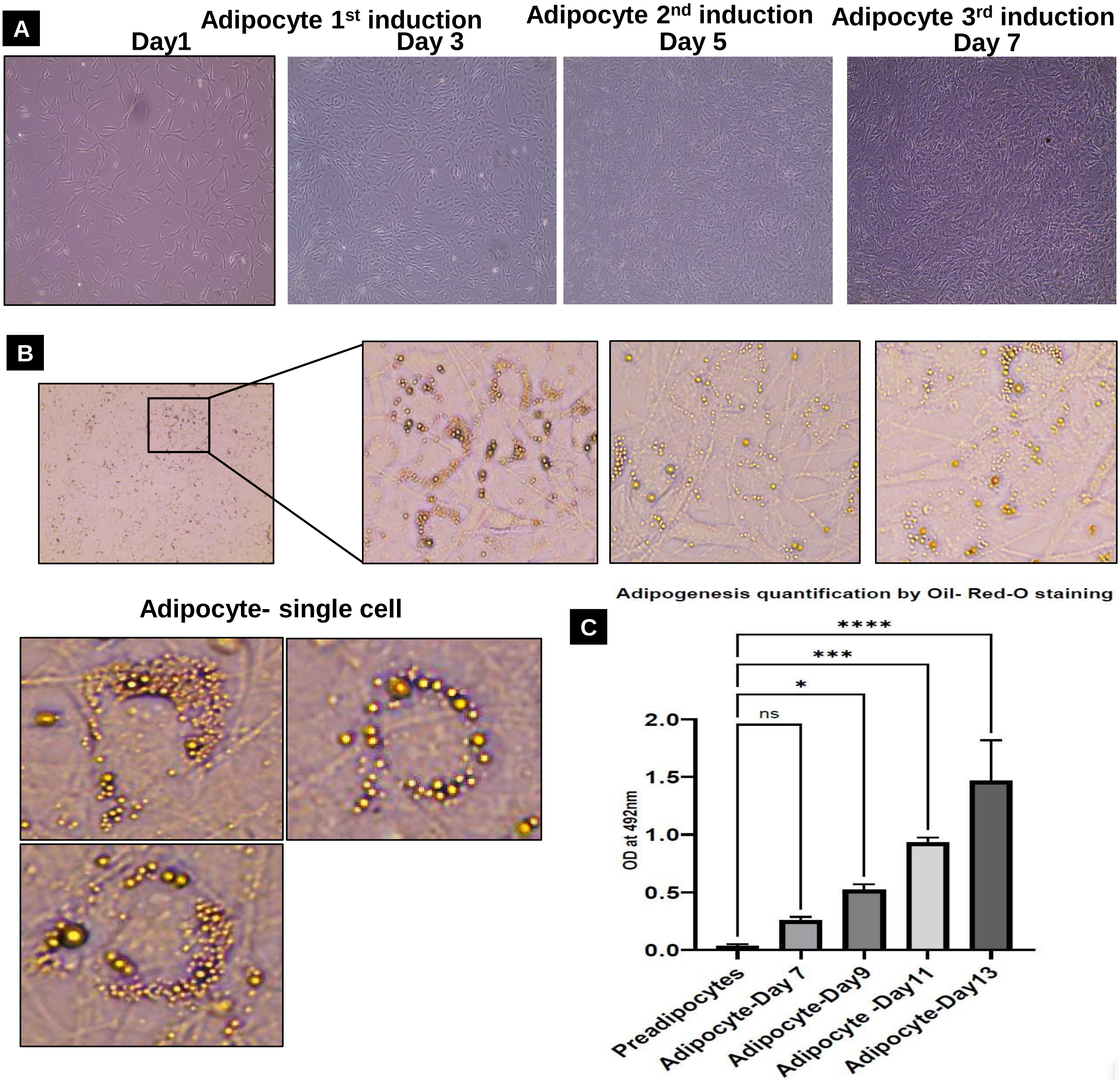

Sup. Fig. 6. Differentiation of adipose derived mesenchymal stem cells to adipocytes :

A. Adipose derived mesenchymal stem cells were cultured till confluency and then were initiated to differentiate into adipocytes 72hrs post confluency on Day3, 5&7 and then continued to be cultured in adipocyte maintenance medium for further differentiation and maturation till day 15. Light (10x)and phase contrast (40x) microscope pictures of cells at different time points of adipocyte differentiation showing increased lipid accumulation.

B & C. Confirmation and Quantification of Adipocyte formation: Cells were grown on a 12 well plate and then fixed and stained with 0.2% oil red O in 2-propanol for 10 min at room temperature at different time points to measure the adipogenesis. After washing, plates were dried, and dye was eluted with 100% 2-propanol, and were transferred to a microtiter plate for reading the extracted dye at 492nm for correlation of adipogenesis.

Sup Figure 7

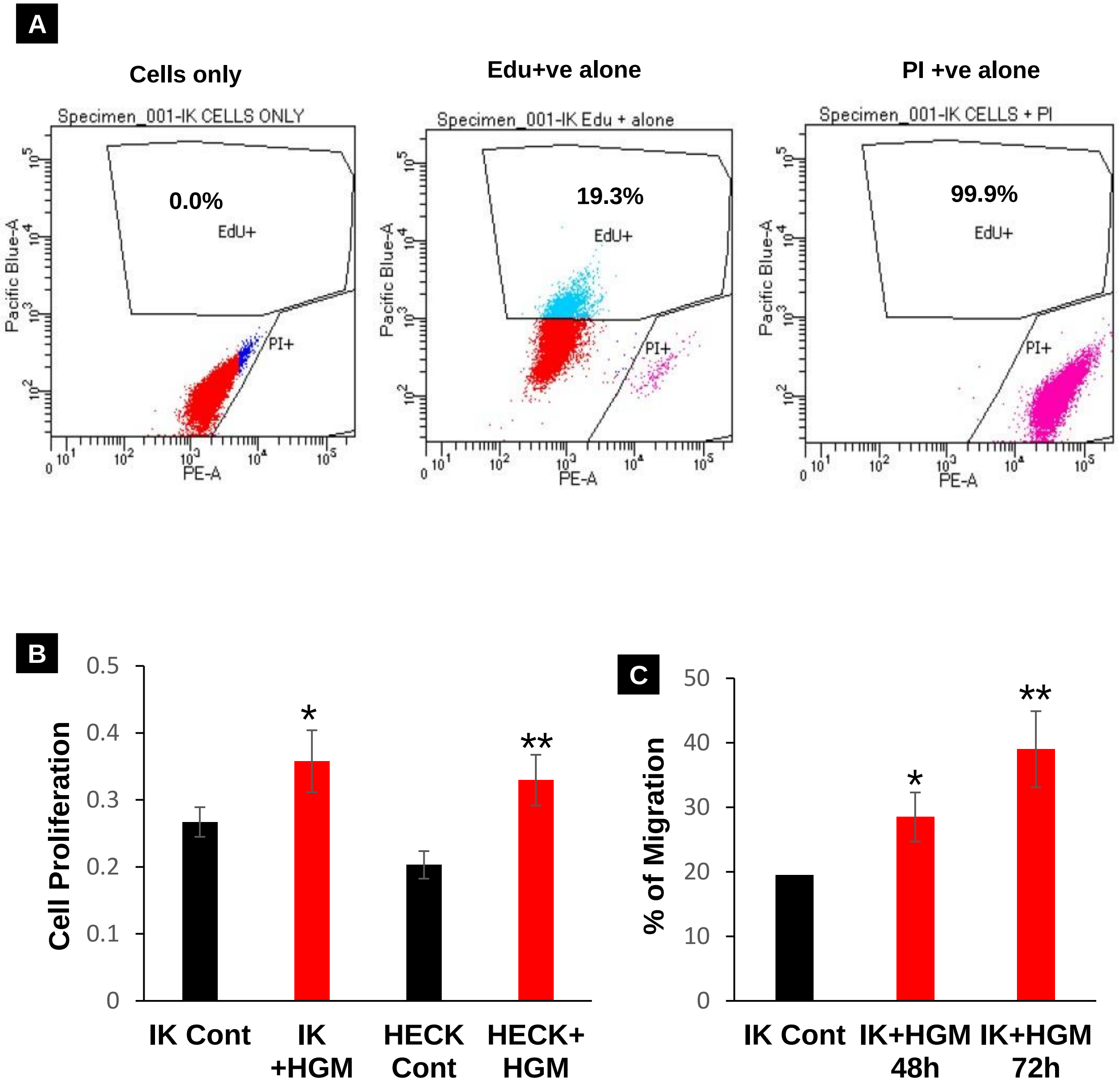

Sup. Figure 7. (A) Cell proliferation controls in IK cells stained with Edu and propidium iodide(PI). **(B & C)** IK or HEC1B cells treated with regular or high glucose (HGM) medium for 24 to 72 hours; cell proliferation measured by EdU staining and migration assessed by wound healing assay (n=4, $p < 0.01$ or 0.005).

Sup Figure 8

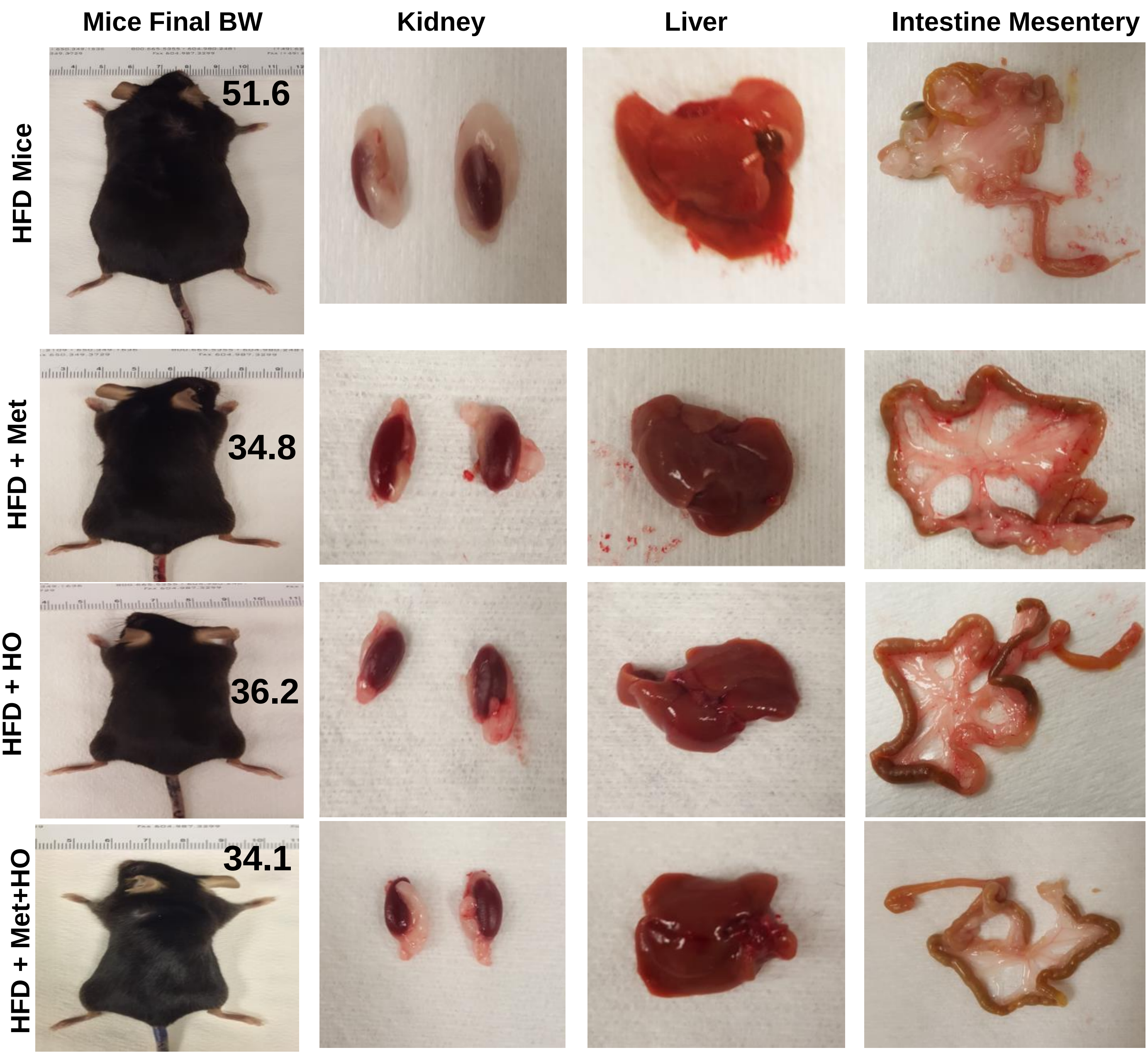

Sup. Fig. 8. Immunocompetent mice were treated with a high-fat diet (HFD, 45% protein calorie) for 24 weeks. Changes in body weight were assessed at the end (24 weeks) of the HFD treatment with small molecule inhibitor HO-3867 (5mg/kg, weekly twice) and Metformin (10mg/kg, daily in water). Representative images depict accumulated levels of adipose tissue in kidney, liver and intestine, which is enlarged in mice fed a HFD for 24 or treatment groups 8 weeks.

Sup Figure 9

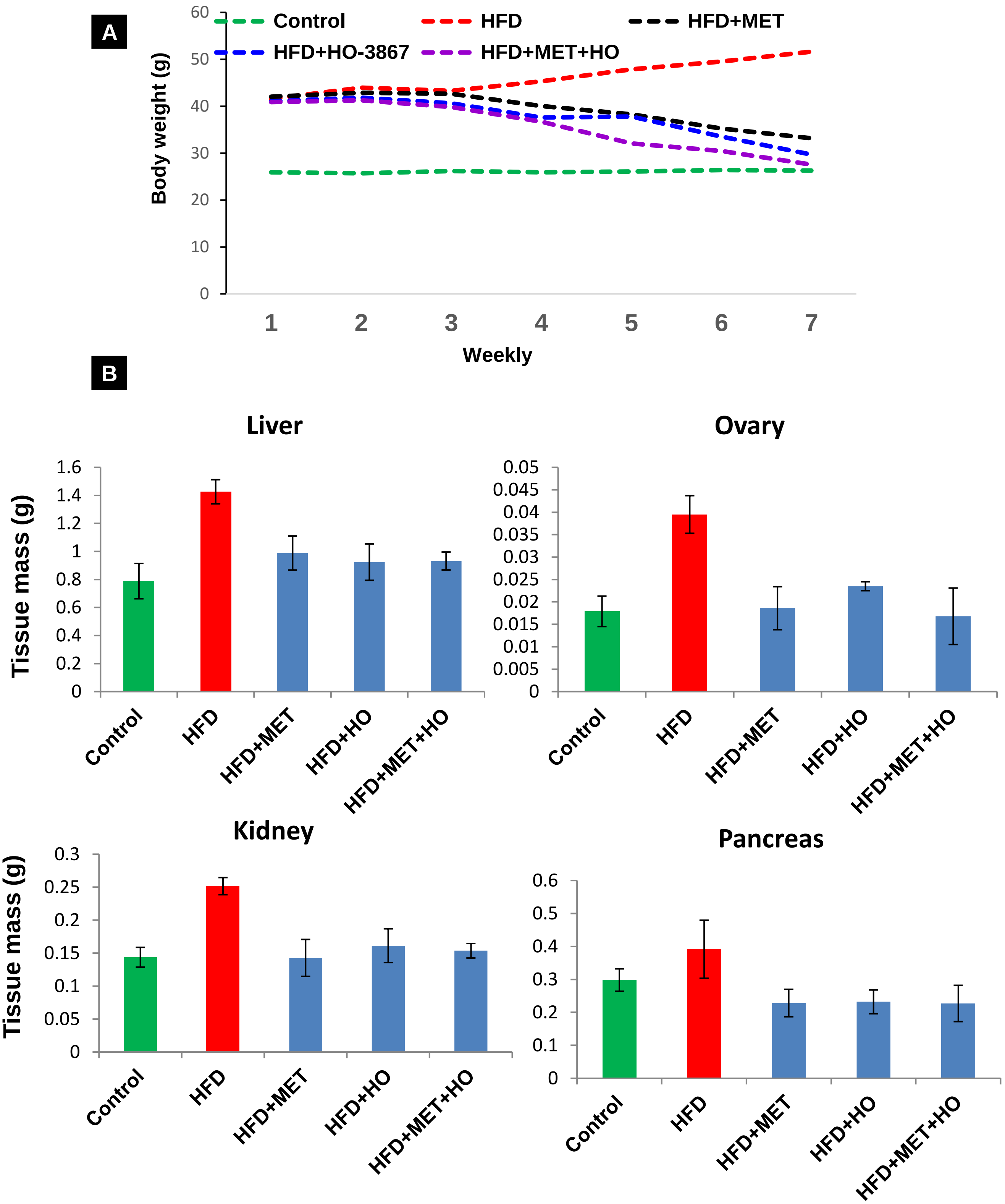

Sup. Fig. 9. A & B. Changes in body, and internal organs liver, kidney, ovary and pancreas weight were assessed at the beginning of first weeks) and end (24 weeks) of the HFD treatment, HO-3867 and Metformin treatment mice (n = 10/group, p<0.05).
